# Vascular response to biolimus A-9 eluting stent in patients with shorter and prolonged dual antiplatelet therapy: optical coherence tomography sub-study of the NIPPON trial

**DOI:** 10.1007/s00380-018-1131-7

**Published:** 2018-02-20

**Authors:** Koji Kuroda, Toshiro Shinke, Hiromasa Otake, Hiroto Kinutani, Raisuke Iijima, Junya Ako, Hisayuki Okada, Yoshiaki Ito, Kenji Ando, Hitoshi Anzai, Hiroyuki Tanaka, Yasunori Ueda, Shin Takiuchi, Yasunori Nishida, Hiroshi Ohira, Katsuhiro Kawaguchi, Makoto Kadotani, Hiroyuki Niinuma, Kazuto Omiya, Takashi Morita, Kan Zen, Yoshinori Yasaka, Kenji Inoue, Sugao Ishiwata, Masahiko Ochiai, Toshimitsu Hamasaki, Kazushi Urasawa, Toru Kataoka, Minoru Yoshiyama, Kenshi Fujii, Takumi Inoue, Masahito Kawata, Hiroyoshi Yokoi, Masato Nakamura, On behalf of the NIPPON investigators

**Affiliations:** 1 Division of Cardiovascular, Department of Cardiology, Kobe University Graduate School of Medicine, 7-5-1 Kusunoki-cho, Chuo-ku, Kobe, Hyogo 650-0017 Japan; 2 Division of Cardiovascular Medicine, Toho University Ohashi Medical Center, Tokyo, Japan; 3 Department of Cardiovascular Medicine, Kitasato University Hospital, Sagamihara, Japan; 4 Department of Cardiology, Seirei Hamamatsu General Hospital, Hamamatsu, Japan; 5 Division of Cardiology, Saiseikai Yokohama-City Eastern Hospital, Yokohama, Japan; 6 Department of Cardiology, Kokura Memorial Hospital, Kitakyushu, Japan; 7 Cardiology Department, Ota Memorial Hospital, Ota, Japan; 8 Department of Cardiology, Tokyo Metropolitan Tama Medical Center, Tokyo, Japan; 9 Cardiovascular Division, National Hospital Organization Osaka National Hospital, Osaka, Japan; 10 Department of Cardiology, Higashi Takarazuka Satoh Hospital, Takarazuka, Japan; 11 Department of Cardiovascular Medicine, Takai Hospital, Tenri, Japan; 12 Department of Cardiology, Edogawa Hospital, Tokyo, Japan; 13 Department of Cardiology, Komaki City Hospital, Komaki, Japan; 14 Department of Cardiology, Kakogawa Central City Hospital, Kakogawa, Japan; 15 Department of Cardiology, St. Luke’s International Hospital, Tokyo, Japan; 16 Division of Cardiology, St. Marianna University School of Medicine Yokohama City Seibu Hospital, Yokohama, Japan; 17 Division of Cardiology, Osaka General Medical Center, Osaka, Japan; 18 Department of Cardiovascular Medicine, Omihachiman Community Medical Center, Omihachiman, Japan; 19 Department of Cardiology, Hyogo Brain and Heart Center, Himeji, Japan; 20 Department of Cardiology, Juntendo University Nerima Hospital, Tokyo, Japan; 21 Division of Cardiovascular Center, Toranomon Hospital, Tokyo, Japan; 22 Division of Cardiology and Cardiac Catheterization Laboratories, Showa University Northern Yokohama Hospital, Yokohama, Japan; 23 Department of Data Science, National Cerebral and Cardiovascular Center, Suita, Japan; 24 Cardiovascular Center, Tokeidai Memorial Hospital, Sapporo, Japan; 25 Department of Cardiovascular Medicine, Bell Land General Hospital, Kyoto, Japan; 26 Cardiovascular Medicine, Osaka City University Hospital, Osaka, Japan; 27 Cardiovascular Center, Sakurabashi Watanabe Hospital, Osaka, Japan; 28 Department of Cardiology, Hyogo Prefectural Awaji Medical Center, Sumoto, Japan; 29 Department of Cardiology, Akashi Medical Center, Akashi, Japan; 30 Department of Cardiovascular Medicine Center, Fukuoka Sanno Hospital, Fukuoka, Japan

**Keywords:** Optical coherence tomography, Dual antiplatelet therapy, Biolimus A9 eluting stent, Intra-stent thrombus

## Abstract

Dual antiplatelet therapy (DAPT) with thienopyridine and aspirin is the standard care for the prevention of stent thrombosis. However, the optimal duration and effect of the duration of DAPT on intra-stent thrombus (IS-Th) formation are unknown. The NIPPON study (Nobori Dual Antiplatelet Therapy as Appropriate Duration) was an open label, randomized multicenter, assessor-blinded, trial designed to demonstrate the non-inferiority of shorter (6-month) DAPT to prolonged (18-month) DAPT, after biolimus A9 eluting stent implantation in 3773 patients at 130 sites in Japan. Among them, 101 patients were randomly allocated for an optical coherence tomography (OCT) sub-study to assess the difference of local IS-Th formation between the two groups. In addition to standard OCT parameters, the number of IS-Th formed was counted in each target stent at 8 months. Baseline patient characteristics were not different between the 6- and 18-month groups. IS-Th was detected in 9.8% of the cases and the presence of IS-Th was not significantly different between the two groups (10.9% in 6-month vs. 9.1% in 12-month, *P* = 0.76). Furthermore, the number of IS-Th formed was not significantly different between the two groups. This OCT sub-study was in line with the main NIPPON study which demonstrated the non-inferiority of 6-month DAPT to 18-month DAPT. Shorter DAPT duration did not promote progressive IS-Th formation at the mid-term time point.

**Electronic supplementary material:**

The online version of this article (10.1007/s00380-018-1131-7) contains supplementary material, which is available to authorized users.

## Introduction

Dual antiplatelet therapy (DAPT) with thienopyridine and aspirin is the standard of care for prevention of stent thrombosis [1]. Previous guidelines recommended that DAPT should be continued for at least 12 months in all patients undergoing drug-eluting stent (DES) implantation [2]. However, recent studies have reported that stopping DAPT earlier in selected patients with DES was as safe and efficient as prolonged DAPT [3–8]. Thus, the optimal duration of DAPT remains controversial and the impact of DAPT duration on local vascular reaction has not been elucidated.

NIPPON (Nobori Dual Antiplatelet Therapy as Appropriate Duration) is an open label, randomized multicenter, assessor-blinded, trial designed to demonstrate the non-inferiority of shorter (6-month) DAPT to prolonged (18-month) DAPT after biolimus A9 eluting stent (BES) implantation. This NIPPON main study demonstrated that 6 months of DAPT was not inferior to 18 months of DAPT following implantation of BES in terms of net adverse clinical and cerebrovascular events (all-cause mortality, myocardial infarction, stroke, and major bleeding) [9]. Among the enrolled patients, 101 patients were randomly allocated for an optical coherence tomography (OCT) sub-study to assess the local vascular responses to BES implantation. The aim of this study was to compare local vessel healing and in-stent thrombus formation between patients with shorter (6-month) and prolonged (18-month) DAPT.

## Materials and methods

### Patient population

NIPPON is an open-label, randomized, multicenter, assessor-blinded trial designed to demonstrate the non-inferiority of shorter (6-month) DAPT to prolonged (18-month) DAPT after NOBORI stent implantation in patients with coronary artery disease from December 2011 to June 2015 at 130 Japanese institutions (supplementary Appendix 1). Nobori (Terumo Corporation, Tokyo, Japan) is a biolimus A9-eluting stent (BES) with an abluminal-side biodegradable polymer coating that degrades 6–9 months after stent implantation [10]. During hospitalization for percutaneous coronary intervention (PCI), the patients were assigned to 6 or 18 months of DAPT at a 1:1 ratio by central randomization using an interactive web-based system. This study was designed to approximate an all-comers trial with broad inclusion criteria to reflect the real-world clinical setting, and patients with acute myocardial infarction (MI) were also enrolled. The extremely limited exclusion criteria were in-stent restenosis (bare metal stent or DES) and index PCI for saphenous vein graft disease or unprotected left main trunk disease. The inclusion and exclusion criteria are detailed in supplementary Appendix 2. The study protocol was approved by the institutional review board at each participating center. Written informed consent was obtained from all patients. The study was conducted in accordance with the Declaration of Helsinki and was registered at Clinical Trial Registration (NCT.01514227). A total of 3773 patients were enrolled to NIPPON trial. Among them, 101 patients at 14 Japanese institutions (supplementary Appendix 3) were randomly allocated for OCT sub-study to assess deference of local in-stent thrombus (IS-Th) formation between two groups at 8–12 months after BES implantation.

### Dual antiplatelet therapy

All patients were receiving aspirin (81–162 mg/day). Patients also received ticlopidine (200 mg/day) or clopidogrel (75 mg/day) for the assigned duration after PCI. The allowance of DAPT duration in the two groups was defined as NIPPON trial protocol. The following per-protocol analysis was performed in this OCT sub-study: patients in the 6-month DAPT group were referred for analysis when the DAPT had been stopped until at least 1 month before the follow-up OCT procedure. In the 18-month DAPT group, DAPT had been continued at the time of the OCT follow-up procedure.

### OCT examination

The follow-up OCT examination was performed 8–12 months after BES implantation. The frequency-domain OCT system (C7 Dragonfly™ or C8 Dragonfly™; St. Jude Medical, St. Paul, MN, USA) was used in the present study. OCT examination was performed, as previously reported [11]. In the use of the frequency-domain OCT system, a 0.014-inch standard guide wire was positioned distally in the target vessel and the frequency OCT catheter was advanced to the distal end of the target lesion. The entire length of the region of interest was scanned using the integrated automated pullback device at 10 or 20 mm/s. For image acquisition, blood in the coronary artery was replaced with iodine contrast media and continuously flushed.

### OCT analysis

**Fig. 1:**
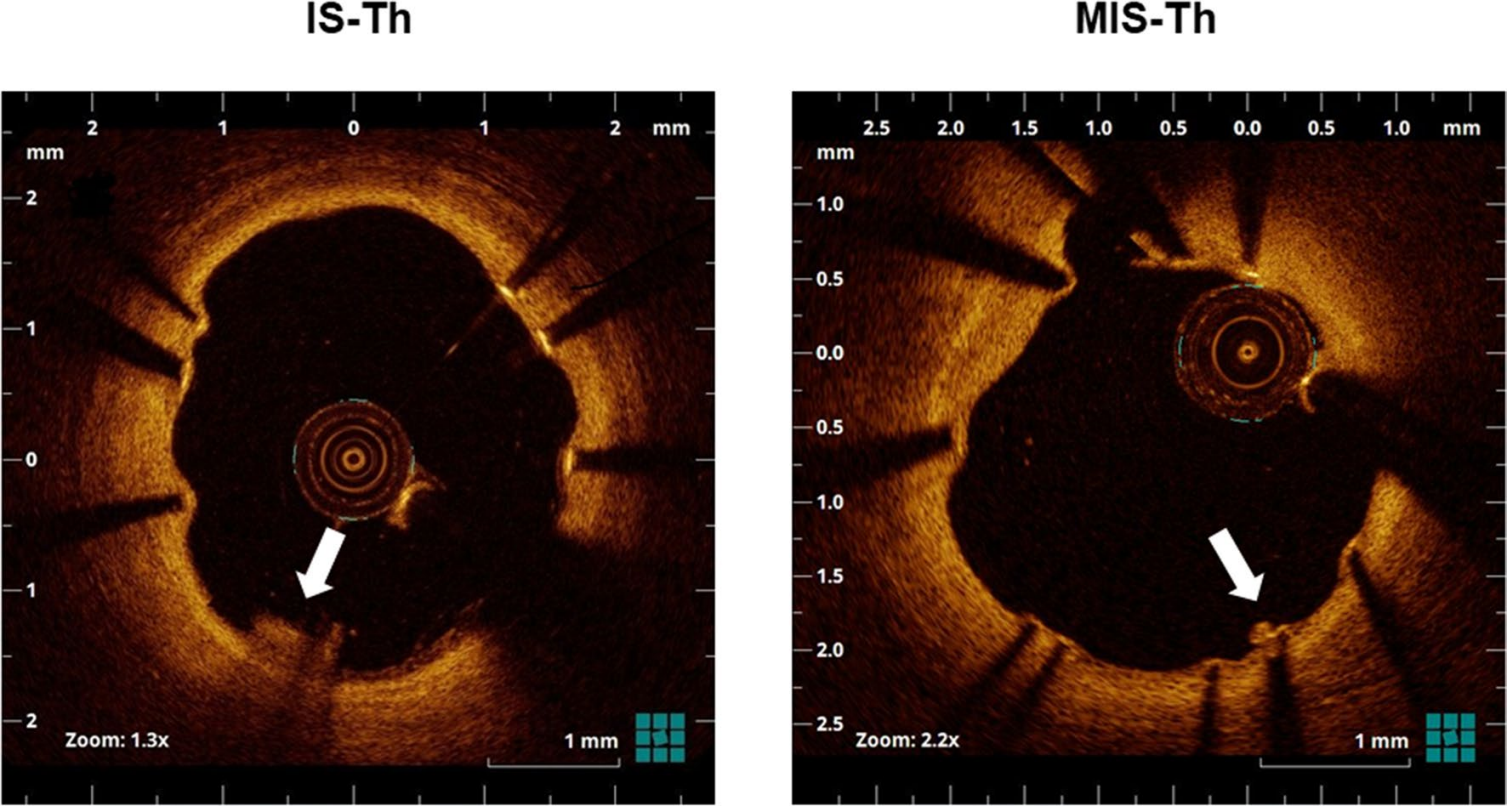
Representative optical coherence tomographic images of IS-Th and MIS-Th (arrow). IS-Th was defined as a mass protruding beyond the stent strut into the lumen, with significant attenuation behind the mass with a height over 250 µm. MIS-Th was defined as a mass with a height of 100–250 μm. *IS-Th* in-stent thrombus, *MIS-Th* micro in-stent thrombus

Off-line OCT analysis was performed using a dedicated software (LightLab Imaging, Westford, MA, USA). All images were analyzed at every frame in stents by independent investigators, who were blinded to the angiographic and clinical findings. For quantitative analysis, cross-section OCT images were analyzed at 1-mm intervals. As qualitative analysis, IS-Th was defined as a mass protruding beyond the stent strut into the lumen, with significant attenuation behind the mass with a height greater than 250 µm [12, 13] (Fig. 1). In this present study, we additionally counted the mass with a height of 100–250 μm as micro-in-stent thrombus (MIS-Th) for the detailed assessment of small IS-Th (Fig. 1). Stent struts were classified as uncovered if any part of the strut was visibly exposed to the lumen or covered if a layer of tissue was visible all over the reflecting surfaces. Neointimal thickness was measured from the center reflection of the stent strut to the vessel-lumen border (neointimal surface or strut surface if uncovered) for each stent strut. An uncovered strut was defined as a strut with a neointimal thickness equal to 0 μm [14]. The frequency of covered and uncovered struts was calculated as the number of those struts divided by the total number of struts for each stent. A malapposed strut was defined as a distance greater than 140 µm between the center reflection of the strut to the vessel wall [15]. The presence of peri-strut low-intensity area (PLIA) was counted per stent. PLIA was defined as a region around stent struts with a homogeneous lower-intensity appearance than surrounding tissue without significant signal attenuation behind the area [16].

### Statistical analysis

Continuous variables were shown as mean ± SD and comparisons between two groups were performed using two-sample *t* test or Wilcoxon rank sum test. Discrete variables were presented as frequencies and percentages, and comparisons were performed by Chi-square test or Fisher’s exact test. All *P* values were 2-sided, and *P* < 0.05 was considered to indicate statistical significance. The statistical analysis was conducted using the commercially available SPSS software version 23 (SPSS, Chicago, IL, USA).

Statistical sample size calculation was not done for this sub-study. This is because there was no past report assessing the difference of local IS-Th formation between shorter and prolonged DAPT duration. We enrolled the largest possible number of cases into the OCT sub-study from NIPPON study enrollment.

## Results

### Patient and lesion characteristics

**Fig. 2:**
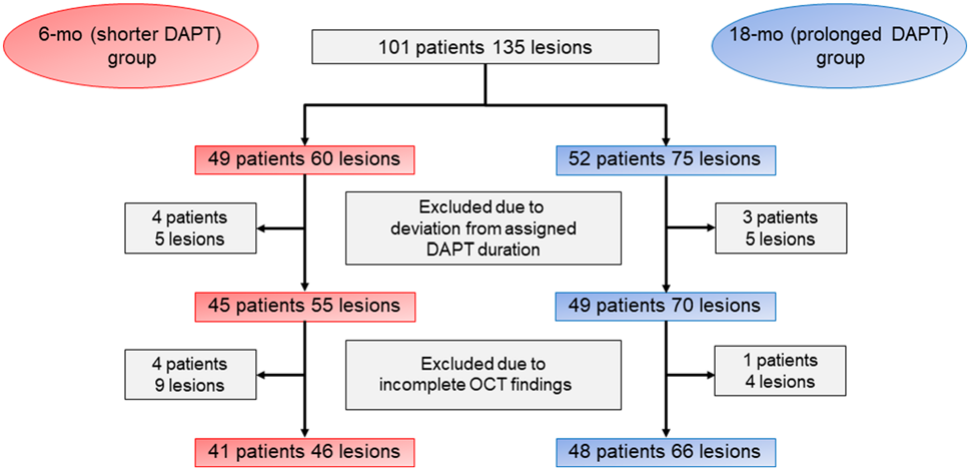
Study population

Among 101 patients (135 lesions), 12 patients (23 lesions) were excluded [7 patients (10 lesions), due to deviation from assigned DAPT duration; 5 patients (13 lesions), due to incomplete OCT examination]. Therefore, 89 patients (112 lesions) were enrolled. A total of 41 patients (46 lesions) and 48 patients (66 lesions) were assigned to the 6- and 18-month groups, respectively (Fig. 2). There were no significant differences between the two groups in terms in patient, medication, laboratory data, and lesion characteristics at the OCT follow-up (Tables 1, 2).

**Table 1:** Baseline clinical characteristics

Variable	6-month (*n* = 41)	18-month (*n* = 48)	*P* value
Clinical characteristics			
Age (years)	67.1 ± 10.1	66.6 ± 9.0	0.80
Male	27 (65.9)	36 (75.0)	0.34
Diabetes mellitus	16 (39.0)	23 (47.9)	0.40
Hypertension	34 (82.9)	31 (64.6)	0.052
Dyslipidemia	28 (68.3)	32 (66.7)	0.87
Current smoker	10 (24.4)	13 (27.1)	0.77
Angina status			0.97
STEMI	4 (9.8)	4 (8.3)	1.00
Stable AP	21 (51.2)	23 (47.9)	0.76
Unstable AP	9 (22.0)	13 (27.1)	0.58
SMI	6 (14.6)	7 (14.6)	0.99
Past medical history			
PCI history	11 (26.8)	9 (18.8)	0.36
BMS implantation	8 (19.5)	6 (12.5)	0.37
DES implantation	4 (9.8)	3 (6.3)	0.70
CABG history	0 (0.0)	2 (4.2)	0.50
Cerebral infarction	0 (0.0)	1 (2.1)	1.00
TIA	1 (2.4)	0 (0.0)	0.46
Intracranial bleeding	0 (0.0)	0 (0.0)	–
Gastric ulcer bleeding	1 (2.4)	1 (2.1)	1.00
Atrial fibrillation	0 (0.0)	1 (2.1)	1.00
PAD	3 (7.3)	0 (0.0)	0.09
Medication			
NSAIDs	1 (2.4)	2 (4.2)	1.00
Beta blocker	16 (39.0)	13 (27.1)	0.23
ARB	20 (48.8)	25 (52.1)	0.76
ACE-I	6 (14.6)	2 (4.2)	0.14
Ethyl icosapentate	1 (2.4)	2 (4.2)	1.00
PPI	24 (58.5)	31 (64.6)	0.56
Steroid	1 (2.4)	0 (0.0)	0.46
Statin	32 (78.0)	40 (83.3)	0.53
Laboratory data			
Total cholesterol (mg/dL)	173.0 ± 30.2	167.4 ± 31.2	0.44
HDL-cholesterol (mg/dL)	54.0 ± 12.0	52.9 ± 15.6	0.72
LDL-cholesterol (mg/dL)	96.1 ± 24.5	95.1 ± 23.3	0.86
Hb_A1C_ (%)	6.09 ± 0.63	6.50 ± 1.19	0.06
Creatinine (mg/dL)	0.85 ± 0.18	0.83 ± 0.22	0.76

Values are presented as mean ± SD or absolute numbers (%)

*ACE-I* angiotensin converting enzyme inhibitor, *AP* angina pectoris, *ARB* angiotensin receptor blocker, *BMS* bare metal stent, *CABG* coronary artery bypass graft, *DES* drug-eluting stent, *NSAIDs* nonsteroidal anti-inflammatory drugs, *PAD* peripheral artery disease, *PCI* percutaneous coronary intervention, *PPI* proton pump inhibitor, *SMI* silent myocardial ischemia, *STEMI* ST elevation myocardial infarction, *TIA* transient ischemic attack

**Table 2:** Baseline lesion characteristics

Variable	6-month (*n* = 46)	18-month (*n* = 66)	*P* value
Duration between PCI and OCT follow-up (days)	293.2 ± 49.9	278.0 ± 41.4	0.093
Lesion location			0.35
Left anterior descending artery	30 (65.2)	34 (51.5)	
Left circumflex artery	7 (15.2)	13 (19.7)	
Right coronary artery	9 (19.6)	19 (28.8)	
Stent size (mm)	3.04 ± 0.40	3.08 ± 0.34	0.55
Stent length (mm)	23.4 ± 12.7	26.6 ± 14.9	0.24

Values are presented as mean ± SD or absolute numbers (%)

*OCT* optical coherence tomography, *PCI* percutaneous coronary intervention

### OCT characteristics

Stent, lumen, and neointimal characteristics had no significant difference between the two groups in OCT findings. Except in stent struts characteristics, the percentage of uncovered struts was significantly higher in the 6-month group (Table 3).

**Table 3:** Optical coherence tomography findings

Variable	6-month (*n* = 46)	18-month (*n* = 66)	*P* value
Stent characteristics			
Average stent area (mm^2^)	7.29 ± 2.97	7.46 ± 2.56	0.75
Minimum stent area (mm^2^)	5.89 ± 2.50	5.67 ± 2.30	0.63
Maximum stent area (mm^2^)	8.82 ± 3.60	9.16 ± 3.01	0.60
Average stent diameter (mm)	2.98 ± 0.58	3.03 ± 0.51	0.67
Minimum stent diameter (mm)	2.49 ± 0.54	2.43 ± 0.52	0.52
Maximum stent diameter (mm)	3.51 ± 0.74	3.64 ± 0.65	0.33
Lumen characteristics			
Average lumen area (mm^2^)	6.84 ± 2.94	6.85 ± 2.54	0.99
Minimum lumen area (mm^2^)	5.27 ± 2.60	5.03 ± 2.37	0.61
Maximum lumen area (mm^2^)	8.61 ± 3.51	8.69 ± 2.97	0.89
Ratio (min/max lumen area)	0.61 ± 0.15	0.58 ± 0.15	0.33
Neointimal characteristics			
Average neointimal area (mm^2^)	0.50 ± 0.33	0.63 ± 0.36	0.06
% neointimal area (%)	7.97 ± 6.22	9.41 ± 5.35	0.19
Average neointimal thickness (mm)	0.08 ± 0.03	0.09 ± 0.03	0.40
Peri-strut law intensity area (%)	19 (41.3)	37 (56.1)	0.12
Stent struts characteristics			
Total no of cross sections (*n*)	25.0 ± 12.8	27.3 ± 13.7	0.38
Total no of struts (*n*)	252.4 ± 138.7	277.1 ± 187.2	0.45
No of uncovered struts (*n*)	11.57 ± 12.71	8.36 ± 14.85	0.24
% of uncovered struts (%)	4.70 ± 4.92	2.59 ± 2.45	0.009
No of malapposed struts (*n*)	1.13 ± 3.38	0.42 ± 1.27	0.18
% of malapposed struts (%)	0.44 ± 1.20	0.13 ± 0.39	0.10

Values are presented as mean ± SD or absolute numbers (%)

### IS-Th characteristics

**Fig. 3:**
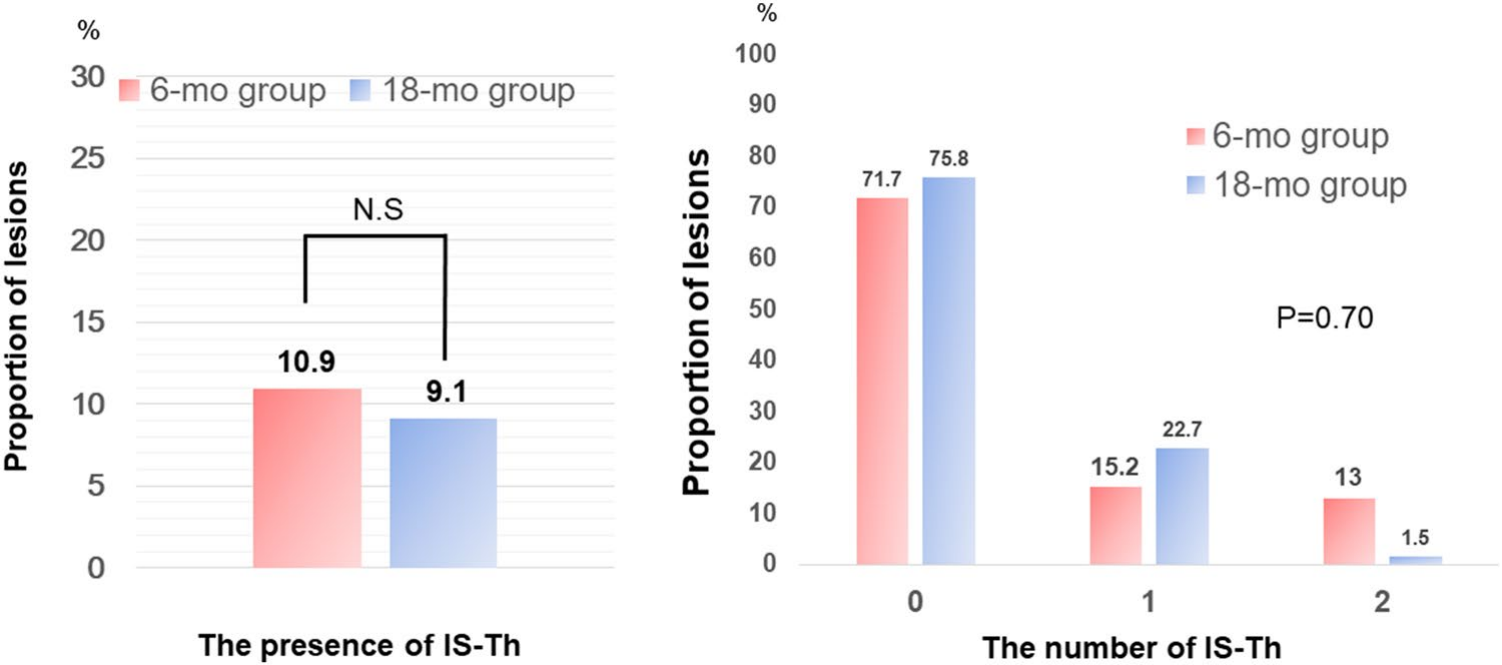
Association between dual antiplatelet therapy duration and in-stent thrombus formation

**Fig. 4:**
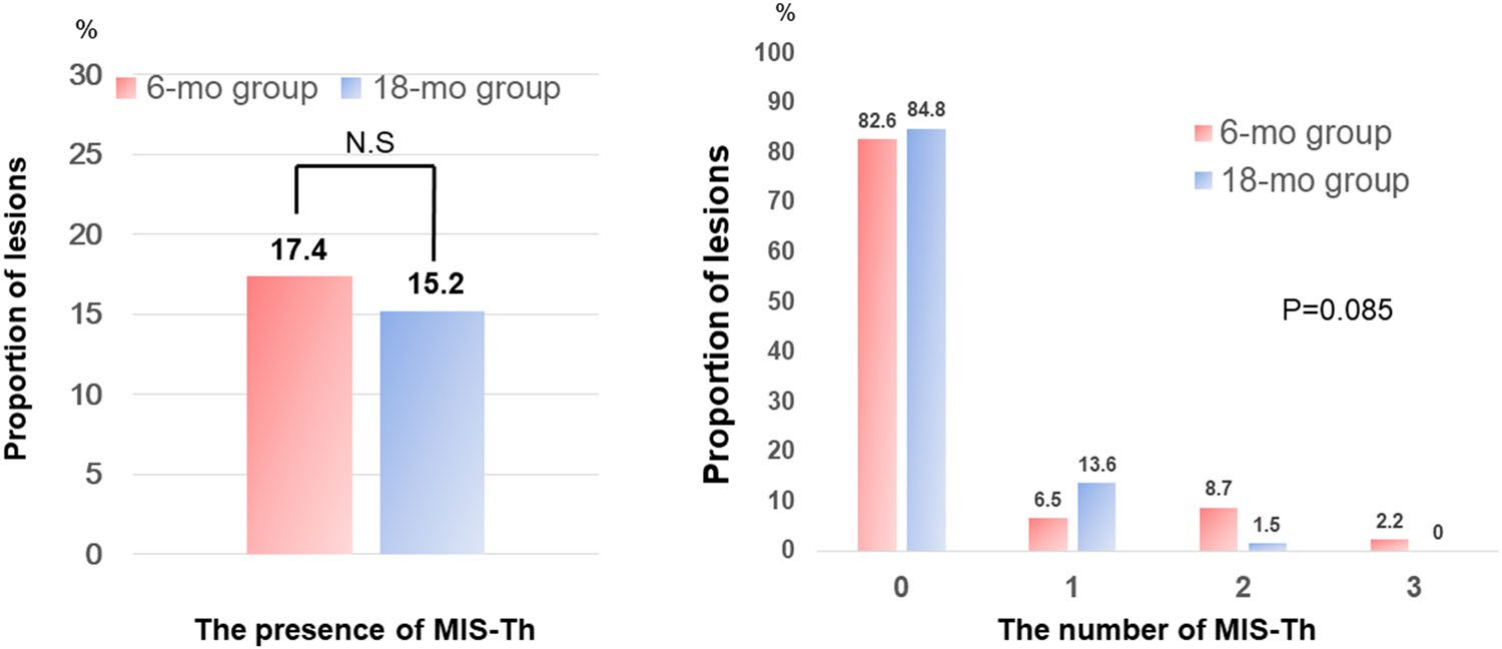
Association between dual antiplatelet therapy duration and micro in-stent thrombus formation

Among 89 patients (112 lesions), IS-Th was identified in 11 lesions (9.8%). The presence and number of IS-Th had no significant difference between the two groups. In addition, the presence and number of MIS-Th had no significant difference between the two groups (Table 4, Figs. 3, 4).

**Table 4:** IS-Th characteristics

Variable	6-month (*n* = 46)	18-month (*n* = 66)	*P* value
IS-Th			
The presence of thrombus	5 (10.9)	6 (9.1)	0.76
The number of thrombi			0.70
0	41 (89.1)	61 (92.4)	
1	4 (8.7)	5 (7.6)	
2	1 (2.2)	0 (0.0)	
MIS-Th			
The presence of thrombus	8 (17.4)	10 (15.2)	0.75
The number of thrombi			0.085
0	38 (82.6)	56 (84.8)	
1	3 (6.5)	9 (13.6)	
2	4 (8.7)	1 (1.5)	
3	1 (2.2)	0 (0.0)	

Values are presented as absolute numbers (%)

*IS-Th* in-stent thrombus, *MIS-Th* micro in-stent thrombus

## Discussion

The NIPPON OCT sub-study was a multicenter, randomized control trial to evaluate the difference of IS-Th formation between shorter and prolonged DAPT duration after BES implantation and was first to demonstrate that the incidence of IS-Th formation at mid-term follow-up was equivalent between the two groups. BES has an abluminal-side biodegradable polymer coating that degrades 6–9 months after stent implantation [10]. Several studies reported that BES had similar stent coverage and apposition and low rate of stent thrombosis as compared to everolimus-eluting stent at mid-term after stent implantation [17, 18].

The frequency of IS-Th at mid-term OCT findings after BES implantation was reported in several studies. The range of the frequency was 7–10% in those studies [19, 20]. In the current study, 11 lesions (9.8%) had IS-Th, and the rate corresponded to these previous studies. This study also showed that the presence and number of IS-Th had no significant difference between the two groups. This result may support previous studies that demonstrated the non-inferiority of short-term DAPT [3–8]. Konishi et al. [19]. has reported that patients with BES implantation achieved favorable vessel healing at 6 months without delayed adverse reaction for up to 12 months.

In the current study, uncovered strut was incidentally higher in the 6-month group than in the 18-month group, but the percentage of malapposed strut was not different. A previous autopsy study evaluating first-generation DESs have shown that an uncovered strut could be a substrate for thrombus formation, which can potentially lead to a thrombotic event [21]. However, a recent clinical trial using OCT for assessing vascular response to DESs has suggested that an uncovered strut, as marker of incomplete vessel healing, may not be directly associated with adverse clinical outcome in second-generation DESs [20]. Regarding BES, stopping DAPT at 6 months does not appear to pose a high risk of provoking IS-Th due to favorable vessel healing during the early phase after stent implantation.

The latest American College of Cardiology/American Heart Association guideline, which is focused on the update on duration of DAPT in patients with coronary artery disease, recommends that patients with stable ischemic heart disease (SIHD) treated with DAPT after DES implantation should undergo P2Y12 inhibitor therapy with clopidogrel for at least 6 months [22]. Identically, European Society of Cardiology guidelines described that DAPT is indicated for 6 months after DES implantation in patients with SIHD [23]. The present study supports these criteria from the viewpoint of local thrombus formation at mid-term OCT findings.

## Limitations

This study has several limitations. First, this was not a double-blind trial; thus, selection bias cannot be excluded. Second, the present study may be not adequately powered to calculate the difference of IS-Th formation due to limited sample size. Despite our plan to enroll 100 patients undergoing DAPT, only 89 patients were finally enrolled in the present study. This is because of the reduced number of patients and the small difference in the primary end-point between the two DAPT groups in the main NIPPON trial. Third, antiplatelet therapy was limited to clopidogrel and ticlopidine in our study; thus, the use of more potent antiplatelet agents may have led to different conclusions. Fourth, in the present study, follow-up OCT was performed at 8–12 months after stent implantation. Therefore, there was only 2–6 months deference of the DAPT duration between the two groups. Longer duration after the cessation or continuation of DAPT may be required to estimate the impact of DAPT duration on IS-Th formation. Fifth, the result of the present study suggested that shorter DAPT duration did not provoke IS-Th formation, however, this study was underpowered to show the association between IS-Th formation and clinical events due to small sample size. Finally, vascular healing response depends on the underlying plaque morphology before stenting [24]. More specifically, vulnerable plaque characteristics in ACS can significantly influence vascular healing after BES implantation. More information regarding plaque morphology at the index procedure should be required.

## Conclusions

This NIPPON OCT sub-study suggested that a shortened DAPT duration does not appear to provoke IS-Th formation after cessation of one antiplatelet regimen at mid-term OCT follow-up. These results were in line with the main NIPPON study which demonstrated the non-inferiority of 6-month DAPT compared with 18-month DAPT.

## Electronic supplementary material

Below is the link to the electronic supplementary material.

Supplementary material 1 (DOCX 35 kb)
