# Supplementary material for: Vascular response to biolimus A-9 eluting stent in patients with shorter and prolonged dual antiplatelet therapy: optical coherence tomography sub-study of the NIPPON trial
Source: Heart Vessels. 2018 Feb 20;33(8):837–45. doi: 10.1007/s00380-018-1131-7 (PMC6060803; doi:10.1007/s00380-018-1131-7)
Supplement: Supplementary file 1 — Supplementary material 1 (DOCX 35 kb) [file 380_2018_1131_MOESM1_ESM.docx]

**Supplementary Appendix**

**NIPPON OCT sub-study**

**TABLE OF CONTENTS**

1. NIPPON study investigators
2. NIPPON study inclusion and exclusion criteria
3. NIPPON OCT sub-study Investigators
4. NIPPON study organization

**Appendix 1. NIPPON study Investigators**

Listed investigators randomized at least one patient.

Hisayuki Okada Seirei Hamamatsu General Hospital

Yoshiaki Ito Saiseikai Yokohama City Eastern Hospital

Hidehiko Hara Toho University Ohashi Medical Center

Kenji Ando Kokura Memorial Hospital

Hitoshi Anzai Ota Memorial Hospital

Hiroyuki Tanaka Tokyo Metropolitan Tama Medical Center

Yasunori Ueda National Hospital Organization Osaka National Hospital

Shin Takiuchi Higashi Takarazuka Satoh Hospital

Yasunori Nishida Takai Hospital

Hiroshi Ohira Edogawa Hospital

Katsuhiro Kawaguchi Komaki City Hospital

Makoto Kadotani, 　　　　Kakogawa Central City Hospital

Hiroyuki Niinuma 　　　　St. Luke's International Hospital

Kazuto Omiya St. Marianna University School of Medicine Yokohama City Seibu Hospital

Takashi Morita Osaka General Medical Center

Kan Zen Omihachiman Community Medical Center

Yoshinori Yasaka Hyogo Brain and Heart Center

Kenji Inoue Juntendo University Nerima Hospital

Sugao Ishiwata Toranomon Hospital

Masahiko Ochiai, Showa University Northern Yokohama Hospital

Itaru Takamisawa 　　　　 Sakakibara Heart Institute

Junji Yajima The Cardiovascular Institute

Takayuki Ishihara　　 Kansai Rosai Hospital

Shigeru Nakamura 　　　　Kyoto Katsura Hospital

Kenshi Fujii Sakurabashi Watanabe Hospital

Kazuhiro Ashida Yokohama Shintoshi Neurosurgical Hospital

Hiroshi Ota Itabashi Central General Hospital

Masaaki Okutsu Nozaki Tokushukai Hospital

Masao Oshima Iseikai Hospital

Ken Kongoji St. Marianna University School of Medicine University Hospital

Yasushi Jinno Handa City Hospital

Ryu Shutta Osaka Rosai Hospital

Nobuo Shiode Tsuchiya General Hospital

Tetsuo Oumi National Diaster Medical Center

Tatsuki Doijiri Yamato Seiwa Hospital

Yoshiaki Yokoi Kishiwada Tokushukai Hospital

Takayuki Ogawa The Jikei University Hospital

Keizo Kimura Social Insurance Kinan Hospital

Mitsuru Munemasa National Hospital Organization Okayama Medical Center

Hiroaki Mukawa 　　 Ogaki Municipal Hospital

Kota Komiyama 　　　　Tokyo Metropolitan Hiroo Hospital

Takeshi Suzuki Toyokawa City Hospital

Takumi Inoue Hyogo Prefectural Awaji Medical Center

Takafumi Ueno Kurume University Hospital

Teruyasu Sugano　　　 Yokohama City University Hospital

Jun Yamashita Tokyo Medical University

Yoshio Yasumura 　　　　Osaka Police Hospital

Takayuki Ogawa The Jikei University Kashiwa Hospital

Haruo Kamiya Japan Red Cross Nagoya Daiichi Hospital

Hiroshi Fujita Japan Red Cross Kyoto Daini Hospital

Toshiro Shinke Kobe University Hospital

Kazushi Urasawa 　　　　Tokeidai Memorial Hospital

Shiro Ono Saiseikai Yamaguchi Hospital

Masayoshi Ajioka 　　　　Tosei General Hospital

Jiro Ando 　　　　The University of Tokyo Hospital

Koichi Mizuno St. Marianna University School of Medicine Kawasaki Municipal Tama Hospital

Haruo Hirayama 　　　　Japan Red Cross Nagoya Daini Hospital

Taiki Tojo Kitasato University Hospital

Yuichiro Maekawa 　　　　Keio University Hospital

Tomohiro Kawasaki Shin Koga Hospital

Takayuki Okamura 　　　　Yamaguchi University Hospital

Fumitoshi Toyota 　　　　Chidoribashi Hospital

Yutaka Hikichi, Saga University Hospital

Ichiro Michishita 　　 Yokohama Sakae Kyosai Hospital

Takafumi Yagi Kyoto Okamoto Memorial Hospital

Hiroshi Kamihata 　　　　Kansai Medical University Hirakata Hospital

Naohisa Shindo Niizashiki Central General Hospital

Nobukazu Ishizaka Osaka Medical College Hospital

Takashi Ashikaga Medical Hospital, Tokyo Medical and Dental University

Yukio Ozaki Fujita Health University Hospital

Hisao Hara National Center for Global Health and Medicine

Hiroshi Sakamoto 　　　　Fuji City General Hospital

Kenji Kada, Japan Community Health care Organization: JCHO Chukyo Hospital

Naofumi Doi Nara Prefecture Western Medical Center

Junko Honye Kikuna Memorial Hospital

Hiroyoshi Yokoi Fukuoka Sanno Hospital

Hitoshi Takano Nippon Medical School Hospital

Masahito Kawata　　 Akashi Medical Center

Hidenori Houzawa 　　　　Ayase Heart Hospital

Toru Ozawa Kobe Rosai Hospital

Arifumi Kikuchi Nippon Medical School Musashi Kosugi Hospital

Kazushige Kadota 　　　　Kurashiki Central Hospital

Yoichi Kijima Osaka Saiseikai Nakatsu Hospital

Tomokazu Ikemoto 　　　　Jichi Medical University Hospital

Yoshihisa Shimada Shiroyama Hospital

Kazuhiko Yumoto 　　　　Yokohama Rosai Hospital

Kenji Kawajiri Matsubara Tokusyukai Hospital

Yoichi Nozaki Hokko Memorial Hospital

Masayoshi Sakakibara IMS Katsushika Heart Center

Atsushi Tosaka, Kawakita General Hospital

Shigetaka Noma, 　　　　Saiseikai Utsunomiya Hospital

Yasushi Wakabayashi Seirei Mikatahara General Hospital

Masaharu Okada, 　　　　Shiga Medical Center for Adults

Mizuki Hirose Meirikai Chuo General Hospital

Yuichiro Takagi KKR Takamatsu Hospital

Takuro Takagi Toshiba General Hospital

Katsumi Miyauchi, 　　　　Juntendo University Hospital

Kazuhiko Misu, St. Marianna University School of Medicine, The New Toyoko Hospital

Satoshi Yasuda National Cerebral and Cardiovascular Center

Ryohei Yoshikawa 　　　　Sanda City Hospital

Ichiro Inoue Hiroshima City Hiroshima Citizens Hospital

Minoru Yoshiyama 　　　　Osaka City University Hospital

Toru Masuyama, Hyogo College of Medicine Hospital

Yoshiaki Tomobuchi, Seiyu Memorial Hospital

Seiji Yamazaki Sapporo Higashi Tokushukai Hospital

Kengo Tanabe Mitsui Memorial Hospital

Kenji Wagatsuma 　　　　Toho University Omori Medical Center

Masayuki Kato Maizuru Kyosai Hospital

Kazuya Kawai Chikamori Hospital

Yuji Hamazaki Showa University Hospital

Masakazu Yamagishi Kanazawa University Hospital

Yoshisato Shibata 　　　　Miyazaki Medical Association Hospital

Kouki Watanabe Saiseikai Matsuyama Hospital

Koichi Tachibana Osaka University Hospital

Hiroshi Wada Saitama Medical Center, Jichi Medical University

Kenji Ninomiya Odawara Cardiovascular Hospital

Hiroshi Suzuki Showa University Fujigaoka Hospital

Jiro Yoshioka Japan Red Cross Nagano Red Cross Hospital

Chikara Mori The Jikei University Daisan Hospital

Masahiro Sonoda National Hospital Organization Kagoshima Medical Center

Toru Kataoka Bell Land General Hospital

Hidenobu Terai Kanazawa Cardiovascular Hospital

Yuko Onishi Hiratsuka Kyosai Hospital

Masanao Toma Hyogo Prefectural Amagasaki Hospital

Takeshi Serikawa　　 Saiseikai Fukuoka General Hospital

Yoritaka Otsuka Fukuoka Wajiro Hospital

Shoji Yano Almeida Memorial Hospital

Soichiro Ebisawa Shinshu University Hospital

Hiroaki Takashima　　 Aichi Medical University Hospital

Hideki Shimomura Fukuoka Tokushukai Medical Center

Yoko Kurumatani　 Kofu-Kyoritsu Hospital

Shinjo Sonoda, University of Occupational and Environmental Health

Hiroki Uehara Urasoe General Hospital

**Appendix 2: DAPT Study Inclusion and Exclusion Criteria**

**2.1 Inclusion Criteria**

Patients must have met all of the following criteria to be eligible for treatment in the study:

1. Age > 20 years and <80 years

2. Optimal indication for percutaneous coronary intervention

3. No known contraindications to dual antiplatelet therapy

4. The subject consented to participate and authorized the collection and release of medical information by signing the “Patient Informed Consent Form”. Informed consent was valid for the duration of the trial or until the subject withdrew.

**2.2 Exclusion Criteria**

Patients were excluded from the study if any of the following criteria were met:

1. Cardiogenic shock at the time of PCI
2. A concomitant disease for which a thienopyridine was essential for treatment
3. A history of stent thrombosis
4. Hypersensitivity or allergy to any of the drugs or components in the “Instructions for Use” of the implanted stent.
5. The subject was unable to give informed consent.
6. Ejection fraction <30%
7. Pregnancy
8. Life expectancy < 1year
9. Any active bleeding condition
10. Inability to undergo complete clinical follow-up
11. A coronary artery not eligible for PCI with the Nobori stent
12. Planned surgery necessitating discontinuation of antiplatelet therapy (>14 days) within 18 months following enrollment.
13. Index stent procedure for a saphenous vein graft, in-stent restenosis of DES, or unprotected LMT lesion.
14. A history of intracranial bleeding or ischemic stroke within 6 months before enrollment.
15. DES for another lesion within 6 months prior to index PCI.
16. Bleeding predisposition or history of coagulation abnormality
17. Concurrent enrollment in another study where the primary endpoint has not been reached or the device/drug could affect major endpoint outcomes in either the open label or randomized phases of the NIPPON study. A subject could only be enrolled in the NIPPON Study once.
18. Patients who were unsuitable for enrollment in the attending physician’s opinion.
19. Subjects on long-term warfarin (or similar anticoagulant) therapy who were anticipated to still be on warfarin at the time of randomization.

**Appendix 3. NIPPON OCT sub-study Investigators**

Toshiro Shinke Kobe University Hospital

Kenji Ando Kokura Memorial Hospital

Kazushi Urasawa 　　　　Tokeidai Memorial Hospital

Toru Kataoka Bell Land General Hospital

Hisayuki Okada Seirei Hamamatsu General Hospital

Minoru Yoshiyama 　　　　Osaka City University Hospital

Makoto Kadotani,　　 Kakogawa East City Hospital

Shin Takiuchi Higashi Takarazuka Satoh Hospital

Kenshi Fujii Sakurabashi Watanabe Hospital

Yoshinori Yasaka Hyogo Brain and Heart Center

Takumi Inoue Hyogo Prefectural Awaji Medical Center

Junya Ako　　　　　　　　Kitasato University

Masahito Kawata　　 Akashi Medical Center

Takashi Morita Osaka General Medical Center

**Appendix 4: NIPPON Study Organization**

**1.1 Executive Committee Members**

Masato Nakamura (Toho University, Ohashi Medical Center)

Hiroyoshi Yokoi　（Fukuoka Sanno Hospital）

Junya Ako　 （Kitasato University）

Toshiro Shinke 　 (Kobe University)

Raisuke Iijima (Toho University, Ohashi Medical Center)

**1.2 Advisory Committee**

Shigeru Saito (Shonan Kamakura General Hospital)

Shinsuke Nanto (Nishinomiya Municipal Hospital)

Kazuaki Mitsudo (Kurashiki Central Hospital)

**1.3 Coordinating Investigators**

Masato Nakamura (Toho University, Ohashi Medical Center)

Hiroyoshi Yokoi　 (Fukuoka Sanno Hospital)

**1.4 Independent Data Monitoring Committee (DMC)**

Chairman:

Tetsu Yamaguchi (Toranomon Hospital)

Voting Members:

Takaaki Isshiki (Ageo Central General Hospital)

Hiroyuki Daida (Juntendo University Hospital)

Masakatsu Nishikawa (Mie University Hospital)

**1.5 Clinical Events Committee**

Adjudicators – Interventional Cardiology

Tadanori Aizawa (The Cardiovascular Institute)

Ryuta Asano (Asano Cardiovascular Clinic)

Akira Yamashina (Tokyo Medical University Hospital)

Adjudicators – Neurology

Yasushi Okada (National Organization Kyushu Medical Center)

Adjudicators – Gastroenterological

Takashi Kawai (Tokyo Medical University Hospital)

**1.6 Statisticians Responsible for Final Analysis**

Toshimitsu Hamasaki (National Cerebral and Cardiovascular Center)

**1.7 Data Coordination**

Data Management:　Bel system

IRT/IWT Services: Cenduit, Durham, NC, US

Web-based Data Capture: Oracle Health Sciences, Boston, MA, US
